# The association between cigarette smoking and inflammation: The Genetic Epidemiology Network of Arteriopathy (GENOA) study

**DOI:** 10.1371/journal.pone.0184914

**Published:** 2017-09-18

**Authors:** Martin Tibuakuu, Daisuke Kamimura, Sina Kianoush, Andrew P. DeFilippis, Mahmoud Al Rifai, Lindsay M. Reynolds, Wendy B. White, Kenneth R. Butler, Thomas H. Mosley, Stephen T. Turner, Iftikhar J. Kullo, Michael E. Hall, Michael J. Blaha

**Affiliations:** 1 Ciccarone Center for the Prevention of Heart Disease, Johns Hopkins School of Medicine, Baltimore, United States of America; 2 St. Luke’s Hospital, Department of Medicine, Chesterfield, United States of America; 3 University of Mississippi Medical Center, Division of Cardiology, Jackson, United States of America; 4 Division of Cardiology, University of Louisville School of Medicine, Louisville, United States of America; 5 Department of Medicine, University of Kansas School of Medicine, Wichita, United States of America; 6 Department of Epidemiology and Prevention, Wake Forest School of Medicine, Winston-Salem, United States of America; 7 Jackson Heart Study, Tougaloo College, Jackson, United States of America; 8 University of Mississippi Medical Center, Department of Medicine, Jackson, United States of America; 9 Division of Nephrology and Hypertension, Mayo Clinic, Rochester, United States of America; 10 Department of Cardiovascular Diseases, Mayo Clinic, Rochester, United States of America; National Yang-Ming University, TAIWAN

## Abstract

To inform the study and regulation of emerging tobacco products, we sought to identify sensitive biomarkers of tobacco-induced subclinical cardiovascular damage by testing the cross-sectional associations of smoking with 17 biomarkers of inflammation in 2,702 GENOA study participants belonging to sibships ascertained on the basis of hypertension. Cigarette smoking was assessed by status, intensity (number of cigarettes per day), burden (pack-years of smoking), and time since quitting. We modeled biomarkers as geometric mean (GM) ratios using generalized estimating equations (GEE). The mean age of participants was 61 ±10 years; 64.5% were women and 54.4% African American. The prevalence of smoking was 12.2%. After adjusting for potential confounders, 6 of 17 biomarkers were significantly higher among current smokers at a Bonferroni adjusted p-value threshold (p<0.003). High sensitivity C-reactive protein was the most elevated biomarker among current smokers when compared to never smokers [GM ratio = 1.39 (95% CI: 1.23, 1.57); p <0.001]. Among former smokers, each pack-year of cigarettes smoked was associated with a 0.4% higher serum level of hsCRP [GM ratio = 1.004 (95% CI: 1.001, 1.006); p = 0.002] and each 5-year lapsed since quitting was associated with a 4% lower serum level of hsCRP [GM ratio = 0.96 (95% CI: 0.93, 0.99); p = 0.006]. However, we found no significant association of smoking intensity or burden with biomarkers of inflammation among current smokers. HsCRP appears to be the most sensitive biomarker of inflammation associated with cigarette smoking of those investigated, and could be a useful biomarker of smoking-related injury for the study and regulation of emerging tobacco products.

## Introduction

Cigarette smoking is one of the leading causes of preventable death in the United States, accounting for almost one in three deaths from cardiovascular disease (CVD) and 20 percent of deaths from ischemic heart disease in adults older than 35 years of age [1, 2]. Despite the progress made over the past 50 years to reduce tobacco use [1, 3], there has been an emergence of novel tobacco products, such as electronic cigarettes, that are claimed to be safer alternatives to combustible cigarettes. However, evidence on the potential cardiovascular toxicity of these products remains unclear [3–5]. The US Food and Drug Administration's (FDA) authority for tobacco regulation has now been extended to include all tobacco products including electronic nicotine delivery systems (ENDS), cigars, and hookahs [6]. To assist in the study and subsequent regulation of these products, prior to the availability of long-term cardiovascular event data, there is a need to identify sensitive and specific biomarkers of tobacco-mediated cardiovascular injury that could be used to evaluate safety and toxicity.

Despite the increased likelihood of cigarette smoking coexisting with chronic inflammatory conditions such as COPD and cancers, prior studies have demonstrated that inflammation itself is on the causal pathway linking cigarette smoking to CVD outside of these chronic inflammatory states [7, 8]. Indeed, a high burden of inflammation has been shown to identify smokers at high risk for CVD [9–11]. In a previous study in the Multi-Ethnic Study of Atherosclerosis (MESA) cohort, we identified hsCRP as the most sensitive marker of subclinical cardiovascular injury compared to markers of thrombosis, subclinical myocardial injury, endothelial damage, and vascular function [12]. However, these studies were limited to a few inflammatory biomarkers [9, 10, 12–15]. We therefore sought to comprehensively study the association of smoking with 17 biomarkers representing different domains in the inflammatory cascade leading to atherosclerotic CVD [16].

Inflammatory biomarkers included biomarkers of systemic inflammation, including high sensitivity C-reactive protein (hsCRP) and serum amyloid A (SAA) [17]; cell adhesion molecules involved in the early phase of plaque development, including intercellular adhesion molecule (ICAM), vascular cell adhesion molecule (VCAM), E-selectin and P-selectin [18, 19]; cytokines, including interleukin-6 (IL-6), interleukin-18 (IL-18) and tumor necrosis factor alpha (effects mediated through tumor necrosis factor receptors 1 and 2, TNFR1 & TNFR2) [20–22]; molecules attracting monocytes to developing plaques, including monocyte chemoattractant protein (MCP) [23]; markers of oxidative stress, including myeloperoxidase (MPO) [24] and receptor for advanced glycated endproducts (RaGE) [25]; and finally enzymes involved in vascular remodeling, including matrix metalloproteinase 2 and 9 (MMP-2 and MMP-9) and their inhibitors, tissue inhibitors of metalloproteinase 1 and 2 (TIMP-1 and TIMP-2) [26–28].

## Materials and methods

This study was IRB approved at the University of Mississippi Medical Center, Jackson and Mayo Clinic, Rochester. All participants provided informed consent.

### Study population

The Genetic Epidemiology Network of Arteriopathy (GENOA) study is one of four research networks that form the National Heart, Lung, and Blood Institute (NHLBI) Family Blood Pressure Program (FBPP) [29]. From its inception in 1995, GENOA's long-term objective was to elucidate the genetics of hypertension and its arteriosclerotic and atherosclerotic target-organ damage, involving the heart, brain, kidneys, and peripheral arteries [30]. Two GENOA cohorts were originally established (1995–2000) through sibships in which at least 2 siblings had essential hypertension diagnosed prior to age 60 years. These include non-Hispanic White Americans from Rochester, MN (N = 1583 at the first exam) and African Americans from Jackson, MS (N = 1854 at the first exam). A third Mexican American cohort was also ascertained through diabetic rather than hypertensive sibships. Because the inflammatory markers of interest were not assessed in this sample, the Mexican American cohort is not included in this report. All siblings in the sibship were invited to participate, both normotensive and hypertensive. During the second exam (2000–2005), approximately 80% of participants were re-recruited to undergo physical examination and provided blood samples for measurement of inflammatory biomarkers.

### Assessment of tobacco exposure

Smoking data were collected using questionnaires administered at the first exam and at the time of blood draw during the second GENOA exam. The latter was used in our analysis. Cigarette smoking was self-reported and assessed by status (never, former, current), intensity (number of cigarettes per day) among current smokers, burden (pack-years) among former and current smokers, and time since quitting (in years) among former smokers. We defined current smokers as those who reported smoking at least one cigarette per day during the year prior to exam date. Former smokers were defined as those who reported not smoking for at least a year prior to exam date, but reported smoking at least one cigarette per day in the past. Never smokers were defined as those who denied ever smoking. Pack-years of smoking was calculated by multiplying the number of packs of cigarettes smoked per day by duration of smoking in years. Per study protocol, participants were asked to refrain from smoking at least four hours before clinic visit.

### Measurement of inflammatory biomarkers

#### Blood collection

Blood was collected by venipuncture after an overnight fast and processed using standardized protocols at each collection site. Blood was centrifuged for 10 min at 4°C, aliquoted in 0.5–1 mL volumes of sodium-citrate plasma, EDTA plasma, and serum and stored at -80°C within 2 h of venipuncture. Aliquots of participants from Jackson, MS samples frozen to -80°C were shipped to Rochester, MN overnight on dry ice. Samples were visually inspected for evidence of thawing and then stored at -80°C. For protein measurements, samples were thawed on ice and aliquoted into bar-coded Eppendorf tubes. The new sample aliquots were re-frozen to -80°C until time of testing at which point they were thawed on ice again. Thus, samples from each collection site were exposed to identical numbers of freeze-thaw cycles for a given assay, ensuring that differential sample handling would not contribute to any subsequently noted ethnic differences in protein levels.

#### Technical assay performance

Given the logistical difficulties of measuring a large number of markers in over 2,500 participants, a subset of markers was measured using multiplex assays; IL-6, IL-18, P-selectin, RAGE, TNFRI, E-selectin, MCP-1, MMP-2, MMP-9, TIMP-1, TIMP-2, TNFRII, and ICAM were measured using a contracted service with SearchLight^™^ Technologies (Boston, MA). The remaining proteins were either measured in the investigators’ laboratory or the Mayo Immunochemical Core Laboratory (Rochester, MN). We evaluated intra- and inter-assay imprecision at a minimum of one level for each analyte to assess technical assay performance (please see Table A in S1 File). For analytes measured at Mayo, we prospectively determined intra-assay variability, reported as coefficient of variation (CV), by measuring the candidate protein markers in blood samples from volunteers in 10 parallel measurements. We also measured inter-assay variability by measuring the same samples across 10 assay runs. We retrospectively determined precision for the assays performed by SearchLight^™^ based on data derived from a blinded, internal plasma control sample. Due to plate-to-plate variations in protein levels in the SearchLight^™^ data sets, we developed an algorithm to reduce inter-plate variability; normalized data were used for subsequent analyses [31].

#### Quality control

Our quality control program included evaluation of intra-assay variability between duplicate sample measurements and inter-assay variability of quality control materials. Protein levels were measured in duplicates. Sample measurements with CVs >20% were either retested or excluded from the dataset. Acceptable imprecision of measurements from the SearchLight^™^ platform was set at <30% due to performance limitations; mean values of samples with CVs >30% were replaced with a singlet value closest to the plate median because retesting was not feasible. We monitored inter-assay variability by measuring 1–3 quality control materials as part of each assay run, and we interpreted the results using a multi-rule approach (1_3s_ and 2_2s_ Westgard rules) [32]. These rules reject all data included in an assay run if any level of quality control material was three standard deviations (SD) above or below the target value or if 2 or more levels were 2 SD beyond the target value in the same direction. Except for proteins measured on the SearchLight^™^ platform, acceptable CV between inter-plate measurements was <20% for all assays and analyses were performed in real-time. Two levels of SearchLight^™^ controls and one normal serum control were embedded randomly across study plates and evaluated retrospectively using a modified multi-rule approach as described elsewhere [31]. Sample measurements from failed plates were either repeated or excluded from the data set.

### Measurement of covariates

Demographics and medical history were collected from standardized questionnaires. Height was measured by stadiometer and weight by electronic balance to calculate body mass index (BMI) (kg/m^2^). Resting systolic and diastolic blood pressure (BP) levels were measured with a random zero sphygmomanometer in the right arm. The diagnosis of hypertension was based on either BP measurements (systolic BP≥140 or diastolic BP ≥90 mmHg), previous diagnosis of hypertension, or current treatment with anti-hypertensive medications. Diabetes was considered present if a participant had fasting serum glucose levels ≥126 mg/dL or was receiving treatment with insulin, oral hypoglycemic agents. Information about physical activity, the use of alcohol, BP medications, statins and aspirin was obtained from questionnaires completed by the participants. Serum cholesterol, high-density lipoprotein (HDL) cholesterol, glucose and creatinine were measured by standard enzymatic methods. The Mayo Clinic Quadratic equation was used to estimate glomerular filtration rate (eGFR) [33].

### Statistical analyses

A total of 2759 participants (1518 from Jackson, MS and 1241 from Rochester, MN) returned for the second GENOA visit. We excluded 57 participants for missing smoking and baseline confounding variables, resulting in 2,702 participants in our analytical sample. Baseline characteristics of the study population were summarized by categories of smoking status. Data were summarized using counts (proportions) for categorical variables, and means (standard deviations) or medians (interquartile ranges) for continuous variables. Chi-squared test, ANOVA, and Kruskal-Wallis testing were used for comparison across smoking categories where appropriate.

First, all biomarkers were ln-transformed to approximate a normal distribution and we imputed for missing biomarkers (range: 5.9% for hsCRP to 33.9% for MMP-9) (Table A in S1 File) using multiple imputations by chained equations (MICE). Our imputation model included all biomarkers and all our baseline confounding variables. By default, MICE imputed for missing biomarkers in order of increasing missingness using data available for other biomarkers and all confounding variables [34]. Second, to study the association between cigarette smoking and inflammatory biomarkers, we used generalized estimating equations (GEE) that account for intra-familial correlations within GENOA [35]. To compare biomarkers, beta coefficients from GEE models were exponentiated to express geometric mean (GM) ratios [36] of each biomarker with smoking status, intensity, burden, and time since quitting. Models were adjusted for demographics (age, sex, race, education); lifestyle variables (alcohol use, physical activity); other traditional CVD risk factors (body mass index, diabetes, systolic blood pressure, total cholesterol, high density lipoprotein cholesterol), estimated glomerular filtration rate, and medication use (antihypertensives, lipid lowering medications, aspirin). We tested for effect modification by race/ethnicity and sex using multiplicative interaction terms, because plasma levels of several of these biomarkers in this population have been previously shown to differ significantly by these factors. [16]

All statistical analyses were performed using Stata 13 (StataCorp LP, College Station, TX) and statistical significance was generally considered at *P* value<0.05. To reduce the chances of obtaining false positive results associated with simultaneously testing 17 biomarkers, a statistical significant level was set at a Bonferroni adjusted P-value of <0.003 (0.05/17).

## Results

### Baseline characteristics of participants

Baseline characteristics of the study population stratified by categories of smoking status are presented in Table 1. The mean age of study participants was 61 ±10 years; 64.5% were women and 54.4% African American. Current smokers constituted 12.2% of the sample and they were likely to be younger (58±10 years), African American (61.2%), currently consume alcohol (65.5%), and use antihypertensive medications (57.0%).

**Table 1:** Baseline characteristics of study participants by categories of smoking status (2000–2005).

Variable	Overall Population	Never Smoker	Former Smoker	Current Smoker	*P* value^a^
Number	2,702	1,503 (55.6)	871 (32.2)	328 (12.1)	NA
Age (years)	61 ± 10	61 ± 10	61 ± 9	58 ± 10	<0.001
Male	967 (35.5)	362 (23.9)	462 (52.6)	143 (43.6)	<0.001
African American	1482 (54.4)	887 (58.4)	394 (44.9)	201 (61.2)	<0.001
Education (yrs)	12 (12–14)	12 (12–16)	12 (12–14)	12 (11–14)	<0.001
Current alcohol use	1431 (52.6)	682 (45.0)	534 (60.9)	215 (65.5)	<0.001
Physical activity index	11 (9–14)	11 (9–14)	11 (8–14)	11 (9–14)	0.561
Body mass index (kg/m^2^)	31 ± 7	32 ± 7	32 ± 6	29 ± 7	<0.001
Systolic blood pressure (mmHg)	135 ± 20	136 ± 20	134 ± 18	133 ± 22	0.002
Antihypertensive use	1913 (70.3)	1068 (70.5)	658 (74.9)	187 (57.0)	<0.001
Diabetes	621 (22.8)	324 (21.4)	225 (25.6)	72 (22.0)	0.069
Estimated glomerular filtration rate (ml/min)	93.3 (85.6–101.7)	92.5 (85.3–99.7)	94.0 (84.4–103.5)	97.3 (90.2–108.1)	<0.001
Total cholesterol (mg/dL)	200 ± 39	204 ± 40	194 ± 37	198 ± 39	<0.001
High density cholesterol (mg/dL)	55 ± 17	57 ± 17	52 ± 16	54 ± 19	<0.001
Lipid lowering medication use	686 (25.2)	323 (21.3)	293 (33.4)	70 (21.3)	<0.001
Aspirin use	987 (36.5)	510 (33.9)	375 (43.1)	102 (31.1)	<0.001
Estrogen use	520 (19.2)	327 (21.8)	147 (16.9)	46 (14.0)	<0.001
Pack-years of cigarettes smoked	N/A	N/A	18 (7, 35)	24 (13, 39)	N/A
Number of cigarettes per day	N/A	N/A	N/A	11 (8, 20)	N/A
Smoking duration (years)	N/A	N/A	23 (14, 33)	39 (31, 47)	N/A
Time since quitting (years)	N/A	N/A	18 (10, 27)	N/A	N/A

^a^P-values for continuous variables were calculated using one-way ANOVA or Kruskal-Willis test where appropriate and for categorical variables using chi-square test. Results are reported as means (standard deviations), medians (interquartile ranges), or counts (proportions).

### Inflammatory biomarkers and smoking status

**Fig 1:**
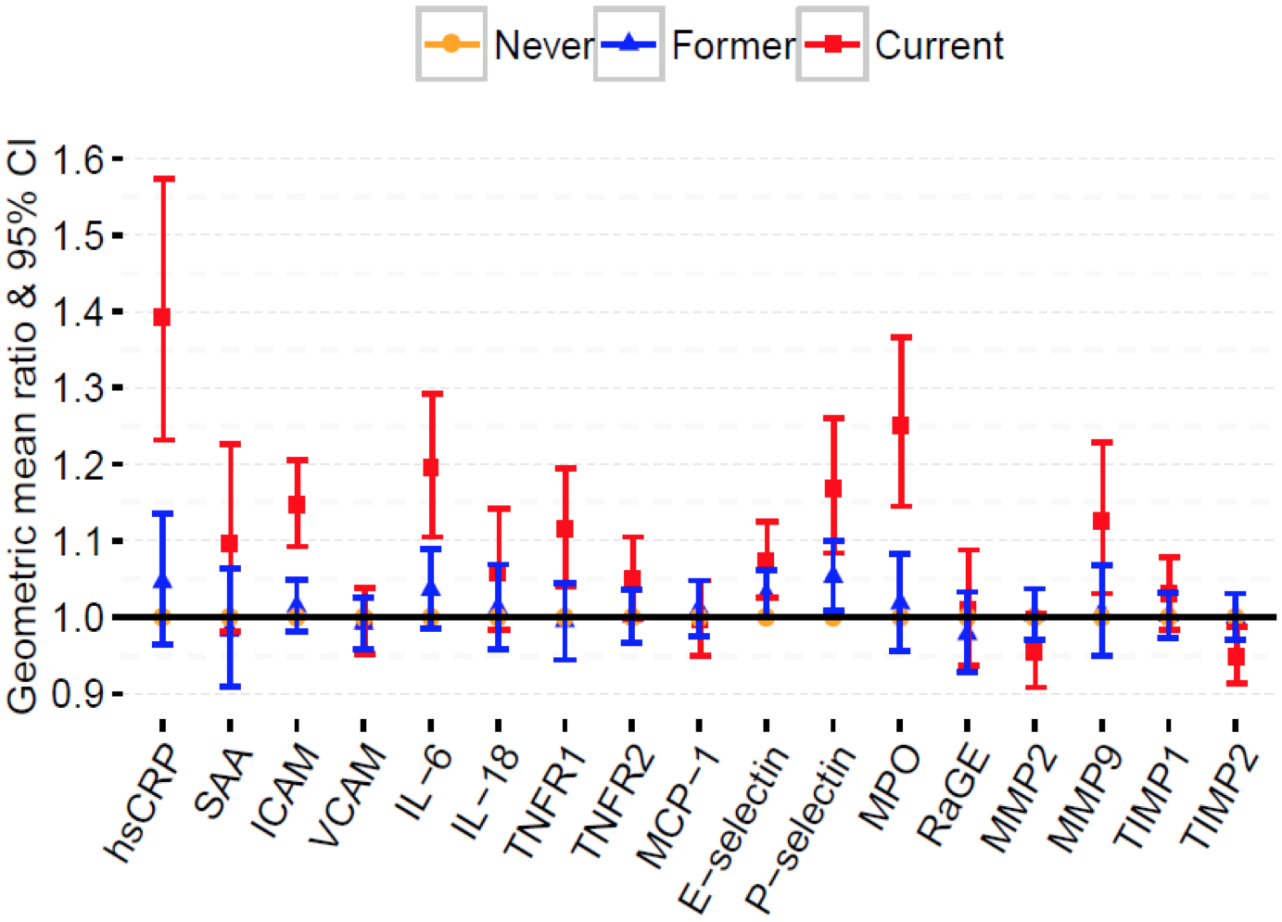
Adjusted geometric mean (GM) ratios with 95% CI of inflammatory biomarkers for current and former smokers vs. referent never smokers. Model adjusted for age, sex, education, race/ethnicity, alcohol use, body mass index, physical activity, estimated glomerular filtration rate, systolic blood pressure, diabetes status, total cholesterol, high density cholesterol, family history of myocardial infarction, antihypertensive use, lipid lowering medication use, aspirin use.

Median plasma concentrations of most inflammatory biomarkers differed significantly by categories of smoking status (Table 2). After adjusting for potential confounders, hsCRP, ICAM, P-selectin, IL-6, TNFR1, and MPO were significantly higher among current smokers at a Bonferroni adjusted p-value threshold (p<0.003), while the following biomarkers did not reach the multiple testing threshold, but were nominally significant (p<0.05): E-selectin, MMP9, and TIMP-2 (Fig 1; Table B in S1 File). Of note, TIMP-2 was lower among current smokers compared to never smokers (p = 0.01). Using a common scale for comparison (GM ratios or percent change), hsCRP was the most elevated biomarker among current smokers when compared to never smokers [GM ratio = 1.39 (95% CI: 1.23, 1.57); p <0.001] (Fig 1; Table B in S1 File).

**Table 2:** Unadjusted baseline distributions of inflammatory biomarkers by categories of smoking status.

Domain of inflammation	Biomarker, units	Overall population	Never smoker	Former smoker	Current smoker	P value^a^
**Systemic Inflammation**						
	HsCRP, mg/L	3.0 (1.4–6.1)	3.0 (1.4–6.2)	2.8 (1.4–5.7)	3.3 (1.7–6.8)	**0.019**
	SAA, μg/mL	19.0 (10.9–33.7)	19.8 (11.8–35.4)	18.3 (10.4–31.0)	17.1 (9.5–33.2)	**0.009**
**Cell adhesion molecules**						
	ICAM, ng/mL	279 (234–331)	274 (231–321)	278 (231–330)	322 (260–397)	**<0.001**
	VCAM, ng/mL	623 (503–760)	622 (510–756)	632 (508–776)	597 (476–727)	**0.017**
	E-selectin, ng/mL	70.0 (57.3–86.4)	68.0 (56.0–84.4)	72.3 (58.6–88.1)	73.4 (58.5–90.1)	**<0.001**
	P-selectin, ng/mL	31.0 (22.7–41.3)	29.4 (21.3–38.9)	32.5 (24.1–42.4)	35.5 (25.7–48.6)	**<0.001**
**Cytokines**						
	IL-6, pg/mL	7.2 (5.2–10.6)	6.9 (5.1–10.1)	7.4 (5.2–10.8)	7.8 (5.6–12.5)	**0.005**
	IL-18, pg/mL	67.7 (47.5–94.9)	66.1 (46.3–93.9)	68.9 (48.3–95.2)	68.7 (49.8–97.2)	0.24
	TNFR1, pg/mL	1,160 (857–1546)	1,145 (865–1490)	1,186 (862–1620)	1165 (809–1662)	0.28
	TNFR2, pg/mL	1,764 (1415–2234)	1,744 (1416–2211)	1,797 (1430–2294)	1773 (1384–2196)	0.29
**Chemoattractant**						
	MCP-1, pg/mL	916 (742–1138)	915 (750–1126)	920 (750–1155)	906 (702–1156)	0.56
**Oxidative stress**						
	MPO, ng/mL	30.6 (20.5–46.9)	29.8 (20.5–46.0)	29.5 (19.5–44.5)	37.2 (24.8–58.5)	**<0.001**
	RaGE, pg/mL	500 (334–730)	513 (335–745)	490 (331–713)	477 (335–730)	0.51
**Vascular remodeling**						
	MMP2, ng/mL	1,742 (1395–2174)	1,766 (1425–2178)	1,750 (1413–2195)	1,618 (1260–2121)	**0.003**
	MMP9, ng/mL	31.7 (23.9–43.4)	31.2 (23.7–41.9)	32.0 (23.8–44.5)	35.5 (25.1–49.4)	**0.004**
	TIMP1, ng/mL	75.9 (61.3–95.8)	74.3 (60.0–92.2)	79.6 (64.0–102.8)	74.4 (61.6–94.5)	**<0.001**
	TIMP2, ng/mL	151 (125–181)	152 (129–181)	151 (124–184)	137 (114–174)	**<0.001**

^**a**^P-values were calculated using Kruskal-Willis test; Bolded items are significant. Results are reported as medians (interquartile ranges).

### Inflammatory biomarkers and smoking intensity

**Fig 2:**
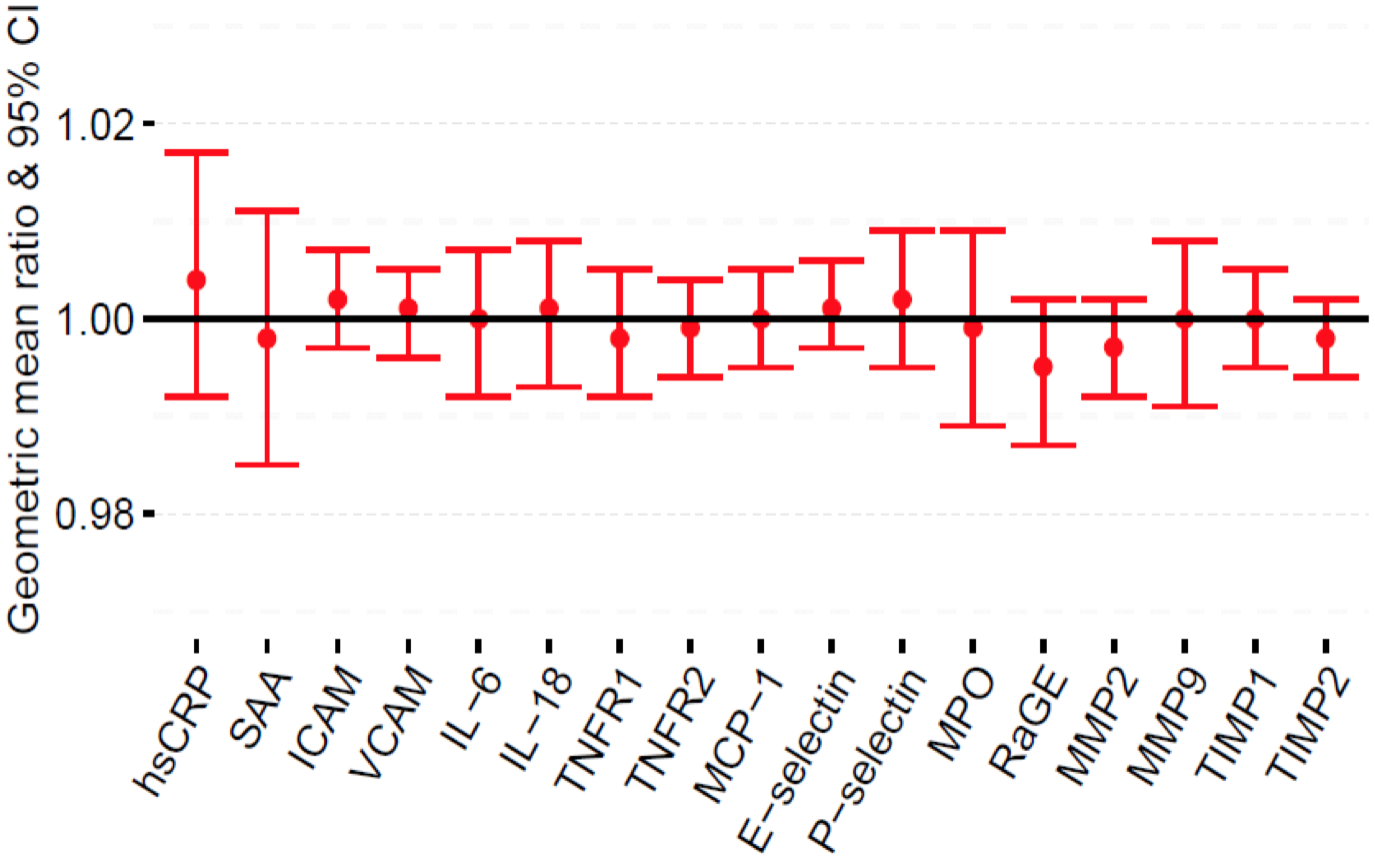
Adjusted geometric mean (GM) ratios with 95% CI of inflammatory biomarkers per unit increase in number of cigarettes smoked/day among current smokers only. Model adjusted for age, sex, education, race/ethnicity, alcohol use, body mass index, physical activity, estimated glomerular filtration rate, systolic blood pressure, diabetes status, total cholesterol, high density cholesterol, family history of myocardial infarction, antihypertensive use, lipid lowering medication use, aspirin use and smoking duration.

Although results were not significant for the association between the number of cigarettes smoked per day and levels of inflammatory biomarkers among current smokers, the point estimate for hsCRP was the most elevated for each cigarette smoked per day after adjustment for multiple confounders (Fig 2; Table D in S1 File).

### Inflammatory biomarkers and smoking burden

**Fig 3:**
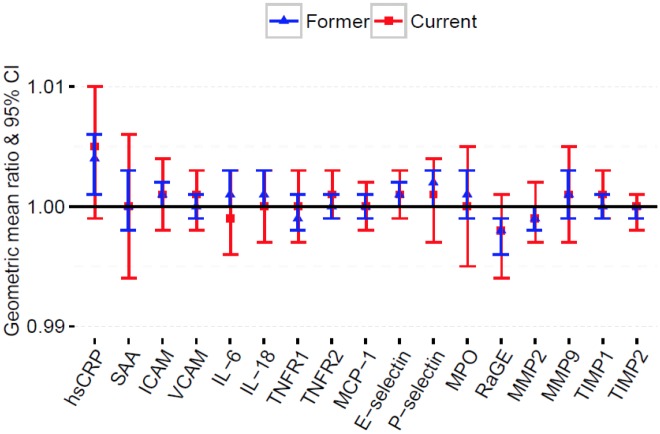
Adjusted geometric mean (GM) ratios with 95% CI of inflammatory biomarkers per unit increase in pack-years of cigarettes smoked among former and current smokers. Model adjusted for age, sex, education, race/ethnicity, alcohol use, body mass index, physical activity, estimated glomerular filtration rate, systolic blood pressure, diabetes status, total cholesterol, high density cholesterol, family history of myocardial infarction, antihypertensive use, lipid lowering medication use, aspirin use.

After multivariable adjustment, smoking burden was not found to be significantly associated with any of our inflammatory biomarkers among current smokers (Fig 3). However, each unit increase in pack-years of smoking was significantly associated with a 0.4% [GM ratio = 1.004 (95% CI: 1.001, 1.006); p = 0.002] higher serum level of hsCRP among former smokers. (Fig 3; Table E in S1 File).

### Inflammatory biomarkers and time since quitting

**Fig 4:**
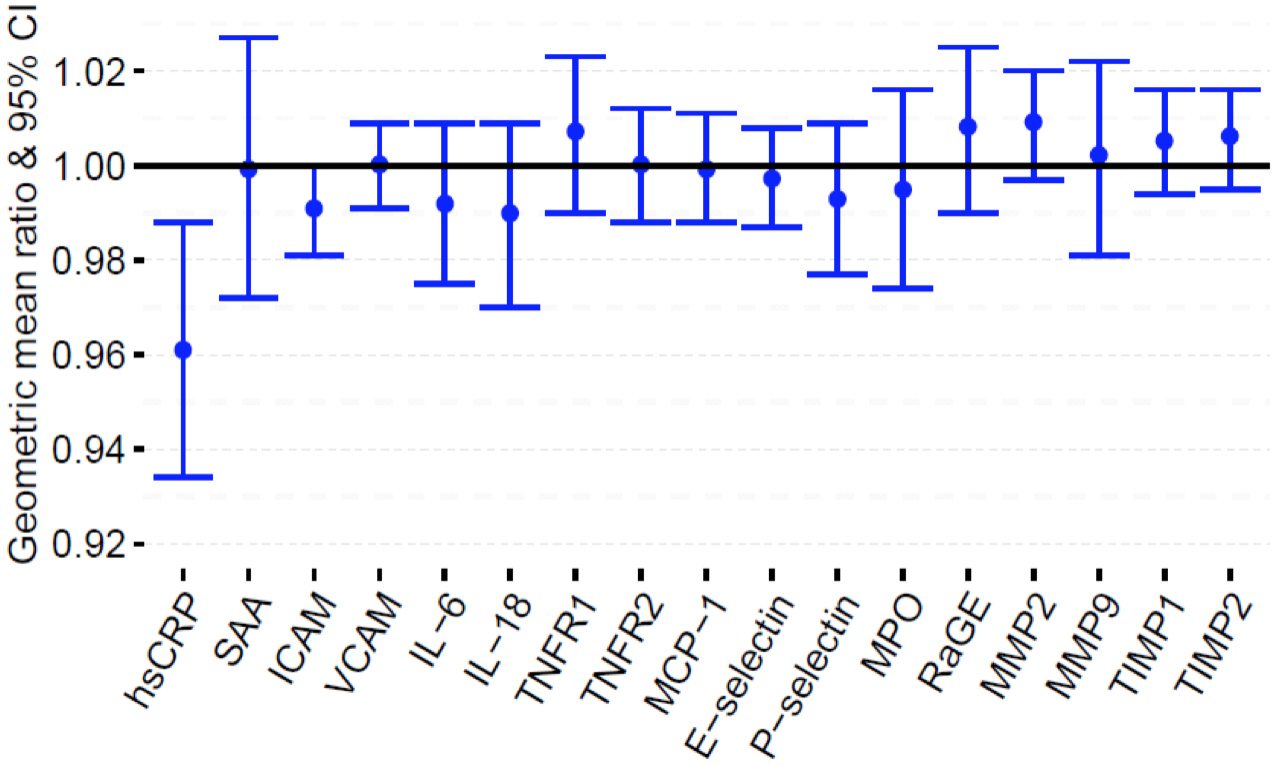
Adjusted geometric mean (GM) ratios with 95% CI of levels of inflammatory biomarkers per 5-year quitting interval. Model adjusted for age, sex, education, race/ethnicity, alcohol use, body mass index, physical activity, estimated glomerular filtration rate, systolic blood pressure, diabetes status, total cholesterol, high density cholesterol, family history of myocardial infarction, antihypertensive use, lipid lowering medication use, aspirin use.

Among former smokers, each 5-year increase in time since quitting was significantly associated with a 4% lower serum level of hsCRP [GM ratio = 0.96 (95% CI: 0.93, 0.99); p = 0.006] after adjusting for multiple variables (Fig 4; Table F in S1 File). Other biomarkers were not significantly associated with time since quitting.

### Effect modification

We observed evidence of interaction by ethnicity (p for interaction = 0.017) but not sex with smoking status. HsCRP for current smokers compared to never smokers was higher among African Americans [GM ratio = 1.51 (95% CI: 1.29, 1.78); p<0.001] than non-Hispanic whites [GM ratio = 1.20 (95% CI: 1.00, 1.43); p = 0.047] (Table C in S1 File).

## Discussion

Among a panel of 17 inflammatory biomarkers that are associated with CVD risk, we demonstrated that current smoking is significantly associated with altered serum levels of 6 biomarkers (hsCRP, ICAM, IL-6, TNFR1, P-selectin and MPO) compared to never smokers at a Bonferroni adjusted p-value threshold (p<0.003). These biomarkers are known to mediate different domains of the inflammatory process of atherosclerotic plaque development, from early endothelial dysfunction to unstable fibrous plaques [37]. For current smokers compared to never smokers, hsCRP was the most elevated for all participants and was even higher among African Americans compared to non-Hispanic whites. Levels of all biomarkers in former smokers were similar to never smokers (Fig 1), highlighting the beneficial effects of smoking cessation on cardiovascular risk factors and disease. While not reaching statistical significance, hsCRP demonstrated the greatest percent elevation with smoking intensity and burden among current smokers. Furthermore, hsCRP was positively associated with smoking burden among former smokers, and inversely associated with time since quitting cigarettes.

Our results are consistent with prior findings that show a relationship between cigarette smoking and vascular inflammation [9–11, 13, 38, 39]. Smoking has been shown to modulate inflammation by activating the NF-_K_B pathway thereby inducing the transcription of genes involved in the innate immune response [40, 41]. Thus, exposure to cigarette smoke produces a complicated systemic inflammatory response through the release of cytokines such as IL-6, which subsequently mediates the release of molecules such as hsCRP and SAA by the liver [40–42]. HsCRP has been identified as a particularly sensitive inflammatory biomarker and a predictor of cardiovascular events [17, 43]. HsCRP has also been shown to be useful in identifying high-risk smokers who may be candidates for intensive smoking cessation programs [10]. The inverse association of time since quitting with hsCRP in this study adds to the growing evidence that vascular inflammation due to smoking may be reversed by long-term smoking cessation and once again highlights the importance of intensive smoking cessation initiatives for high-risk smokers [10, 38]. Conversely, King et al. reported an inverse association of cessation with F_2_ isoprostane:creatinine [F_2_:Cr] ratio and white blood cell (WBC) count but not hsCRP after a year of follow-up among participants who made aided attempt to quit smoking [39]. Although participants had biochemically confirmed 7-day point-prevalence abstinence at 1 year in their study, it is possible that the duration of cessation was not long enough to impact serum levels of hsCRP, which is more distal to F_2_:Cr in the inflammatory cascade.

Surprisingly, we found no significant association of smoking intensity or burden with any of our inflammatory biomarkers among current smokers. These results though conflicting with our previous findings in MESA [12], are consistent with other studies [14, 38, 44, 45]. Shiels et al. also found no association of smoking intensity or burden with serum concentrations of inflammatory markers [38]. Interestingly, similar results were reported by Ohsawa et al. in a multi-center community-based cohort in Japan [44]. The lack of association of smoking intensity and burden with inflammatory biomarkers among current smokers in the present study may be due to the small proportion of current smokers [328/2702 (12.1%)] (Table 1) included in our sample, as evidenced by the wide confidence intervals in Fig 2. Alternatively, the lack of a significant association between smoking intensity or burden with inflammatory biomarkers among current smokers may suggest a low threshold for a ceiling effects of cigarette smoking on these biomarkers [38]. Thus any amount of smoking may lead to alterations in serum levels of these biomarkers, thereby increasing CVD risk and mortality [46].

The results of the present study have important implications for tobacco regulatory science. The goal of our research, which is funded by the American Heart Association Tobacco Regulation and Addiction Center (A-TRAC), a member of the FDA Tobacco Centers of Regulatory Science (TCORS), is to inform the FDA about sensitive biomarkers of subclinical CVD that could be used to study the potential cardiovascular toxicity of novel tobacco products. From our data, we conclude that measuring hsCRP levels among users of electronic cigarettes and comparing these to current combustible cigarette users and non-users may help demonstrate whether novel tobacco products, such as ENDS, carry the same pro-inflammatory potential as traditional cigarette smoking prior to availability of long-term cardiovascular outcomes data.

The main strength of this study is the well-characterized cohort with a comprehensive phenotyping of 17 inflammatory biomarkers of CVD risk. This study expands upon previous studies by including a broad spectrum of inflammatory biomarkers to find the most sensitive biomarker of early cardiovascular injury due to cigarette smoking in a unique cohort that included mostly African Americans predisposed to CVD. Nonetheless, this study has some limitations. First, the cross-sectional design limits our ability to demonstrate causality and investigate changes in concentrations of biomarkers following tobacco exposure. While unlikely, other unaccounted for chronic inflammatory triggers could explain the higher concentrations of inflammatory biomarkers among current smokers. For instance, African-Americans and Non-Hispanic Whites were recruited from different geographic regions and may have had a difference in circulating levels of biomarkers because of difference in environmental exposures not related to smoking. This could explain the ethnic difference in the levels of some of these biomarkers. Second, the analytical precision of the assays varied, and this may have resulted in the lack of significant association between smoking and some biomarkers with poor test performance. Third, although multiple imputations were used to account for missing biomarkers, the high proportion of missingness for some biomarkers (33.9% for MMP-9) could affect the interpretation of these results. Fourth, smoking exposure was self-reported and did not include information on urine cotinine levels to corroborate self-reported smoking behaviors and avoid misclassification of smoking exposure [47]. Finally, we did not assess immediate inhalational exposure to cigarettes in our study and therefore our results can only be applied to epidemiologic studies of novel tobacco products where there is no immediate exposure.

In conclusion, our data support previous findings that biomarkers of inflammation may allow for early detection of cardiovascular injury due to cigarette smoking. HsCRP appears to be the most sensitive biomarker of inflammation associated with cigarette smoking of the 17 biomarkers investigated, although other markers including MPO, ICAM, IL-6, P-selectin, E-selectin and MMP-9 were also elevated in current smokers. In an era when novel smokeless tobacco products are rapidly emerging in the United States and in other parts of the world, hsCRP and potentially other inflammatory biomarkers may be useful for the study and regulation of these products.

## Supporting information

S1 File Supplementary tables (Table A to Table F). (PDF)

S2 File Smoking history questionnaires. (PDF)
