# Supplementary material for: The association between cigarette smoking and inflammation: The Genetic Epidemiology Network of Arteriopathy (GENOA) study
Source: PLoS One. 2017 Sep 18;12(9):e0184914. doi: 10.1371/journal.pone.0184914 (PMC5602636; doi:10.1371/journal.pone.0184914)
Supplement: S1 File — (PDF) [file pone.0184914.s001.pdf]

Supplementary tables

Table A. Biomarkers, Method, Precision of Assay and Percent Missing					
Analyte	Method	Inter-assay imprecision<br><br>CV %, (levels)	Intra-assay imprecision<br><br>CV%, (levels)	Sample matrix	Percent Missing
CRP, mg/L	Immunoturbidometric[1]	1.8-2.6%<br><br>(3.9-10.9 mg/L)	1.0-9.2%<br><br>(0.56-5.2 mg/L)	Serum	5.9
SAA, µg/mL	ELISA[2]	9.0-14.0%<br><br>(15.3-406 µg/mL)	3-11.5%<br><br>(31.3-384 µg/mL)	EDTA plasma	22.0
ICAM, ng/mL	ELISA[3]	11.4-14.1%<br><br>(284-645 ng/mL)	3.6-4.3%<br><br>(324-590 ng/mL)	EDTA plasma	26.0
VCAM, ng/mL	ELISA[3]	5.3-9.8%<br><br>(62.6-868 ng/mL)	4.5-7.3%<br><br>(183-437 ng/mL)	EDTA plasma	7.9
IL-6, pg/mL	ELISA 6-plex[4]	25.6%<br>(4.1 pg/mL)	12.4%<br>(4.6 pg/mL)	EDTA plasma	18.1
IL-18, pg/mL	ELISA 6-plex[4]	29.9%<br>(35.5 pg/mL)	14.3%<br>(35.7 pg/mL)	EDTA plasma	20.7
TNFRI, pg/mL	ELISA 6-plex[4]	29.2%<br><br>(818 pg/mL)	9.4%<br><br>(971.9 pg/mL)	EDTA plasma	21.4
TNFRII, pg/mL	ELISA 9-plex[4]	27.4%<br><br>(1,191 pg/mL)	10.9%<br><br>(1,200 pg/mL)	EDTA plasma	18.0

MCP-1, pg/mL	ELISA 9-plex[4]	32.4% (623 pg/mL)	13.1% (745 pg/mL)	EDTA plasma	20.6
E-selectin, ng/mL	ELISA 9-plex[4]	18.20% (58.8 ng/mL)	8.3% (60.4 ng/mL)	EDTA plasma	17.9
P-selectin, ng/mL	ELISA 6-plex[4]	17.9% (39.7 ng/mL)	7.1% (40.4 ng/mL)	EDTA plasma	19.4
MPO, ng/mL	ELISA[6]	7.2-7.4%, (4.1-166.0 ng/mL)	10.7-12.4% (2.8-52.2 ng/mL)	Serum	12.2
RAGE, pg/mL	ELISA 6-plex[4]	30.1% (1,021 pg/mL)	14.5% (1,038 pg/mL)	EDTA plasma	20.1
MMP-2, ng/mL	ELISA 9-plex[4]	26.9% (1,359 ng/mL)	9.5% (1,398 ng/mL)	EDTA plasma	18.1
MMP-9, ng/mL	ELISA 9-plex[4]	22.6% (155.5 ng/mL)	54.3% (119.2 ng/mL)	EDTA plasma	33.9
TIMP-1, ng/mL	ELISA 9-plex[4]	20.0% (62.4 ng/mL)	5.4% (61.7 ng/mL)	EDTA plasma	18.1
TIMP-2, ng/mL	ELISA 9-plex[4]	25.8% (121.7 ng/mL)	8.0% (121.9 ng/mL)	EDTA plasma	21.8
1. Diasorin, Inc., Stillwater, MN; 2. BioSource International, Camarillo, CA; 3. R&D Systems, Minneapolis, MN, 4. SearchLight™, Pierce, Boston, MA; 5. Calbiochem, Gibbstown, NJ; 6. ALPCO Diagnostics, Salem, NH;					

Table B. Adjusted geometric mean (GM) ratios with 95% CI in levels of inflammatory biomarkers by categories of smoking status.

Domain of inflammation	Biomarker	Never Smoker	Former Smoker		Current Smoker	
			GM Ratio (95% CI)	P value	GM Ratio (95% CI)	P value
**Systemic inflammation**						
	**hsCRP**	REF	1.046 (0.965, 1.135)	0.27	**1.392 (1.232, 1.573)**	**<0.001**
	SAA	REF	0.983 (0.909, 1.064)	0.67	1.097 (0.981, 1.226)	0.11
**Cell adhesion molecule**						
	**ICAM**	REF	1.014 (0.981, 1.049)	0.41	**1.147 (1.092, 1.206)**	**<0.001**
	VCAM	REF	0.992 (0.958, 1.026)	0.64	0.995 (0.952, 1.039)	0.81
	**E-selectin**	REF	1.030 (1.000, 1.061)	0.05	**1.074 (1.026, 1.125)**	**0.003**
	**P-selectin**	REF	**1.053 (1.009, 1.099)**	**0.019**	**1.169 (1.084, 1.260)**	**<0.001**
**Cytokine**						
	**IL-6**	REF	1.036 (0.985, 1.089)	0.17	**1.195 (1.105, 1.292)**	**<0.001**
	IL-18	REF	1.012 (0.958, 1.069)	0.66	1.059 (0.983, 1.142)	0.13
	**TNFR1**	REF	0.995 (0.944, 1.045)	0.84	**1.116 (1.041, 1.195)**	**0.002**
	TNFR2	REF	1.001 (0.967, 1.036)	0.95	1.050 (0.997, 1.105)	0.06
**Chemoattractant**						
	MCP-1	REF	1.011 (0.975, 1.048)	0.56	0.997 (0.949, 1.048)	0.91
**Oxidative stress**						
	**MPO**	REF	1.018 (0.956, 1.083)	0.58	**1.251 (1.145, 1.366)**	**<0.001**
	RaGE	REF	0.978 (0.928, 1.033)	0.43	1.009 (0.936, 1.088)	0.81
**Vascular remodeling**						
	MMP2	REF	1.003 (0.970, 1.037)	0.88	0.955 (0.908, 1.004)	0.07
	**MMP9**	REF	1.007 (0.949, 1.068)	0.83	**1.126 (1.031, 1.229)**	**0.009**
	TIMP1	REF	1.002 (0.972, 1.032)	0.91	1.030 (0.984, 1.079)	0.21
	**TIMP2**	REF	1.000 (0.970, 1.031)	0.99	**0.949 (0.913, 0.988)**	**0.01**

Models adjusted for age, sex, education, race/ethnicity, alcohol use, BMI, physical activity, eGFR, SBP, DM, total cholesterol, HDL, family history of myocardial infarction, antihypertensive use, lipid lowering medication use, aspirin use.

Bolded items indicate nominally significant results.

Table C. Stratified results by ethnicity, for adjusted geometric mean (GM) ratios with 95% CI of inflammatory biomarkers by categories of smoking status.

	Non-Hispanic White					African American			
	Never Smoker	Former Smoker		Current Smoker		Former Smoker		Current Smoker	
**Biomarker**	**REF**	**GM ratio (95% CI)**	**P value**	**GM ratio (95% CI)**	**P value**	**GM ratio (95% CI)**	**P value**	**GM ratio (95% CI)**	**P value**
hsCRP	REF	1.010 (0.906, 1.126)	0.85	**1.199 (1.003, 1.433)**	**0.047**	1.076 (0.953, 1.215)	0.23	**1.514 (1.287, 1.782)**	**<0.001**
SAA	REF	0.990 (0.893, 1.098)	0.85	1.006 (0.843, 1.200)	0.94	0.984 (0.871, 1.112)	0.79	1.155 (0.988, 1.349)	0.07
ICAM	REF	1.028 (0.990, 1.067)	0.15	**1.179 (1.117, 1.244)**	**<0.001**	1.003 (0.949, 1.060)	0.91	**1.121 (1.041, 1.207)**	**0.003**
VCAM	REF	0.999(0.951, 1.048)	0.95	0.962 (0.904, 1.024)	0.22	0.992(0.947, 1.038)	0.72	1.007 (0.951, 1.067)	0.81
IL-6	REF	1.064 (0.987, 1.147)	0.11	**1.204 (1.069, 1.357)**	**0.002**	1.026 (0.957, 1.099)	0.47	**1.177 (1.062, 1.304)**	**0.002**
IL-18	REF	1.012 (0.941, 1.088)	0.74	0.997 (0.883, 1.126)	0.96	1.016 (0.937, 1.102)	0.69	1.092 (0.991, 1.203)	0.08
TNFR1	REF	1.012 (0.941, 1.087)	0.75	**1.127 (1.017, 1.248)**	**0.022**	0.978 (0.910, 1.050)	0.53	1.101 (1.000, 1.213)	0.05
TNFR2	REF	0.999 (0.954, 1.046)	0.95	1.000 (0.928, 1.078)	0.99	1.008 (0.957, 1.062)	0.76	**1.073 (1.002, 1.149)**	**0.045**
MCP-1	REF	1.004(0.960, 1.050)	0.86	1.012 (0.944, 1.085	0.73	1.020 (0.966, 1.078)	0.47	0.982 (0.918, 1.050)	0.59
E-selectin	REF	**1.048 (1.006, 1.092)**	**0.025**	**1.086 (1.018, 1.158)**	**0.012**	1.013 (0.972, 1.056)	0.54	**1.067 (1.000, 1.137)**	**0.049**
P-selectin	REF	**1.083 (1.016 1.154)**	**0.014**	**1.153 (1.030, 1.290)**	**0.013**	1.020 (0.958, 1.086)	0.53	**1.185 (1.082, 1.298)**	**<0.001**
MPO	REF	1.028 (0.937, 1.128)	0.55	**1.341 (1.164, 1.546)**	**<0.001**	1.018 (0.934, 1.108)	0.68	**1.188 (1.058, 1.334)**	**0.004**
RaGE	REF	0.957 (0.889, 1.030)	0.24	0.997 (0.885, 1.124)	0.96	1.005 (0.928, 1.088)	0.91	1.026 (0.930, 1.132)	0.61
MMP2	REF	0.986 (0.941, 1.032)	0.54	0.931 (0.863, 1.004)	0.07	1.019 (0.972, 1.069)	0.43	0.974 (0.910, 1.043)	0.45
MMP9	REF	1.028 (0.946, 1.117)	0.51	**1.157 (1.002, 1.335)**	**0.047**	0.991 (0.916, 1.072)	0.82	**1.085 (0.971, 1.212)**	**0.147**
TIMP1	REF	1.022 (0.980, 1.066)	0.31	1.022 (0.953, 1.097)	0.54	0.984 (0.942, 1.029)	0.48	1.029 (0.964, 1.097)	0.38
TIMP2	REF	0.983 (0.945, 1.023)	0.39	**0.916 (0.860, 0.975)**	**0.006**	1.019 (0.975, 1.065)	0.41	0.976 (0.926, 1.028）	0.36

Model adjusted for age, sex, education, race/ethnicity, alcohol use, BMI, physical activity, eGFR, SBP, DM, total cholesterol, HDL, family history of myocardial infarction, antihypertensive use, lipid lowering medication use, aspirin use.

Bolded items indicate nominally significant results.

Table D. Adjusted geometric mean (GM) ratios with 95% CI of inflammatory biomarkers for each cigarette smoked per day among current smokers.

**Biomarker**	**GM ratio (95% CI)**	**P value**
hsCRP	1.004 (0.992, 1.017)	0.53
SAA	0.998 (0.985, 1.011)	0.79
ICAM	1.002 (0.997, 1.007)	0.41
VCAM	1.001 (0.996, 1.005)	0.74
E-selectin	1.001 (0.997, 1.006)	0.56
P-selectin	1.002 (0.995, 1.009)	0.62
IL-6	1.000 (0.992, 1.007)	0.92
IL-18	1.001 (0.993, 1.008)	0.85
TNFR1	0.998 (0.992, 1.005)	0.61
TNFR2	0.999 (0.994, 1.004)	0.58
MCP-1	1.000 (0.995, 1.005)	0.98
MPO	0.999 (0.989, 1.009)	0.82
RaGE	0.995 (0.987, 1.002)	0.17
MMP2	0.997 (0.992, 1.002)	0.29
MMP9	1.000 (0.991, 1.008)	0.97
TIMP1	1.000 (0.995, 1.005)	0.91
TIMP2	0.998 (0.994, 1.002)	0.41

Model adjusted for age, sex, education, race/ethnicity, alcohol use, BMI, physical activity, eGFR, SBP, DM, total cholesterol, HDL, family history of myocardial infarction, antihypertensive use, lipid lowering medication use, aspirin use and smoking duration.

Bolded items indicate nominally significant results.

Table E. Adjusted geometric mean (GM) ratios with 95% CI of inflammatory biomarkers for each unit increase in pack-years of cigarettes smoked among former and current smokers.

	**Former**		**Current**	
**Biomarker**	**GM ratio (95% CI)**	**P value**	**GM ratio (95% CI)**	**P value**
hsCRP	**1.004 (1.001, 1.006)**	**0.002**	1.005 (0.999, 1.010)	0.12
SAA	1.000 (0.998, 1.003)	0.81	1.000 (0.994, 1.006)	0.91
ICAM	1.001 (1.000, 1.002)	0.26	1.001 (0.998, 1.004)	0.45
VCAM	1.000 (0.999, 1.001)	0.87	1.001 (0.998, 1.003)	0.59
E-selectin	**1.001 (1.000, 1.002)**	**0.024**	1.001 (0.999, 1.003)	0.31
P-selectin	**1.002 (1.000, 1.003)**	**0.032**	1.001 (0.997, 1.004)	0.64
IL-6	1.001 (1.000, 1.003)	0.11	0.999 (0.996, 1.003)	0.77
IL-18	1.001 (1.000, 1.003)	0.08	1.000 (0.997, 1.003)	0.95
TNFR1	0.999 (0.998, 1.001)	0.52	1.000 (0.997, 1.003)	0.85
TNFR2	1.000 (0.999, 1.001)	0.38	1.001 (0.999, 1.003)	0.55
MCP-1	1.000 (0.999, 1.001)	0.64	1.000 (0.998, 1.002)	0.95
MPO	1.001 (0.999, 1.003)	0.19	1.000 (0.995, 1.005)	0.91
RaGE	**0.998 (0.996, 0.999)**	**0.007**	0.998 (0.994, 1.001)	0.19
MMP2	0.999 (0.998, 1.000)	0.06	0.999 (0.997, 1.002)	0.52
MMP9	1.001 (0.999, 1.003)	0.21	1.001 (0.997, 1.005)	0.58
TIMP1	1.000 (0.999, 1.001)	0.92	1.001 (0.999, 1.003)	0.54
TIMP2	1.000 (0.999, 1.000)	0.39	1.000 (0.998, 1.001)	0.62

Model adjusted for age, sex, education, race/ethnicity, alcohol use, BMI, physical activity, eGFR, SBP, DM, total cholesterol, HDL, family history of myocardial infarction, antihypertensive use, lipid lowering medication use, aspirin use.

Bolded items indicate nominally significant results.

Table F. Adjusted geometric mean (GM) ratios with 95% CI of levels of inflammatory biomarkers per 5-year quitting interval

Biomarker	GM ratio (95% CI)	P value
**hsCRP**	**0.961 (0.934, 0.988)**	**0.006**
serum amyloid A	0.999 (0.972, 1.027)	0.96
ICAM	0.991 (0.981, 1.000)	0.05
VCAM	1.000 (0.991, 1.009)	0.96
E-selectin	0.997 (0.987, 1.008)	0.63
P-selectin	0.993 (0.977, 1.009)	0.38
IL-6	0.992 (0.975, 1.009)	0.36
IL-18	0.990 (0.970, 1.009)	0.31
TNFR1	1.007 (0.990, 1.023)	0.44
TNFR2	1.000 (0.988, 1.012)	0.98
MCP-1	0.999 (0.988, 1.011	0.91
MPO	0.995 (0.974, 1.016)	0.63
RaGE	1.008 (0.990, 1.025)	0.39
MMP2	1.009 (0.997, 1.020)	0.13
MMP9	1.002 (0.981, 1.022)	0.88
TIMP1	1.005 (0.994, 1.016)	0.42
TIMP2	1.006 (0.995, 1.016)	0.28

Models adjusted for age, sex, education, race/ethnicity, alcohol use, BMI, physical activity, eGFR, SBP, DM, total cholesterol, HDL, family history of myocardial infarction, antihypertensive use, lipid lowering medication use, aspirin use.

Bolded items indicate nominally significant results.
