# Supplementary material for: The association between cigarette smoking and inflammation: The Genetic Epidemiology Network of Arteriopathy (GENOA) study
Source: PLoS One. 2017 Sep 18;12(9):e0184914. doi: 10.1371/journal.pone.0184914 (PMC5602636; doi:10.1371/journal.pone.0184914)
Supplement: S2 File — (PDF) [file pone.0184914.s002.pdf]

Tobacco Products

1. Have you smoked more than 100 cigarettes in your entire life?

Yes

No — Go to # 2

Other — Go to # 2

comment:______________________

____

1a. In what year or how old were you when you first started smoking?

Year ☐☐☐☐ or Age ☐☐

1b. Do you now smoke cigarettes?

Yes — Go to # 1d

No — Go to # 1c

Other — Go to # 2

comment:______________________

____

1c. In what year or how old were you when you last quit smoking?

Year ☐☐☐☐ or Age ☐☐

1d. On average, how many cigarettes per day do/did you usually smoke?

☐☐☐

2. How many hours per week are you exposed to second hand smoke (because of smoking by others)?

☐☐☐

3. *Any comments regarding participant or completion of the tobacco use form?*

No

Other

comment:______________________

____
